# A new species of winter noctuid moth (Lepidoptera, Noctuidae, Xyleninae) from Zhejiang, China, with a key to species of the genus

**DOI:** 10.3897/BDJ.12.e127939

**Published:** 2024-07-12

**Authors:** Weijun Fang, Yulong Zhang, Min Wang

**Affiliations:** 1 Forestry Bureau of Zhejiang, Chun'an, China Forestry Bureau of Zhejiang Chun'an China; 2 South China Agricultural University, Guangzhou, China South China Agricultural University Guangzhou China; 3 Institute of International Rivers and Eco-Security, Yunnan University, Kunming, China Institute of International Rivers and Eco-Security, Yunnan University Kunming China

**Keywords:** morphology, east China, Xylenini

## Abstract

**Background:**

The tribe Xylenini is one of the main large taxonomic groups known as Winter Noctuidae.

**New information:**

A new species of the genus *Antivaleria* Sugi, 1980, *Antivaleriaronkayorum* Zhang & Wang, **sp. nov.** is described and illustrated from Zhejiang Prov., China. The species resembles *Antivaleriaperegovitsi* Ronkay et al., 2010, but differs in wing pattern, foot-like cucullus and special shape of harpe. A key to the *Antivaleria* species is presented. The holotype is deposited in the Department of Entomology, College of Plant Protection, South China Agricultural University, Guangzhou.

## Introduction

The Asian genus *Antivaleria* was established by Sugi (1980) with *Hadenaviridimacula* Graeser, 1889, as its type species and its characters defined. Ronkay and Ronkay (1995) noted that *munda* Leech, 1900, is a member of *Antivaleria* Sugi, 1980. Afterwards, Hreblay and Ronkay (1997) reported a new species, *Antivaleriaviridentata* Hreblay & Ronkay, 1997 from Taiwan. Subsequently, Kononenko (2001) transferred *munda* to the genus *Atrachea* (see also in Babics et al. (2012)). Most recently, Ronkay et al. (2010) found a new species *Antivaleriaperegovitsi* Ronkay et al., 2010 in Central China.

Before this paper, there were three species recorded. In the present study, we describe a new species similar to *A.peregovitsi* from east China.

## Materials and methods

The specimens were collected using a light trap in China. Adults were photographed by a NIKON CoolPix S7000 digital camera. Abdomens were removed and macerated in hot 10% sodium hydroxide (NaOH) solution for examination of genitalia, photographs of which were taken under a Zeiss SteReo Discovery V.12. Adults and genitalia photos were all processed by Adobe Photoshop CC2018 software. Terminology of adult and genitalia follows Ronkay et al. (2010).

## Taxon treatments

### Antivaleria ronkayorum

Zhang & Wang sp. nov.

6F477317-DCF9-5AF9-9690-042F228486CB

C1F3EEF5-7F4A-412A-B598-57DBB1E240AD

#### Materials

- **Type status:** Holotype. **Occurrence:** recordedBy: Min Wang & Yining Chen; sex: male; occurrenceID: 9DE2A386-C052-5638-BBD4-FE47627DB287; **Location:** country: China; stateProvince: Zhejiang; county: Chun'an; locality: Tianping Village; verbatimElevation: 1150 m; **Event:** eventDate: 28-10-2023
- **Type status:** Paratype. **Occurrence:** recordedBy: Min Wang & Yining Chen; sex: 3 males, 1 female; occurrenceID: 6C2F22F9-F02A-567B-BD07-581F6C114E97; **Location:** country: China; stateProvince: Zhejiang; county: Chun'an; verbatimElevation: 1050m; **Event:** eventDate: 15-10-2023

#### Description

**Male** (Fig. 1a). Wingspan 38-43 mm, fore-wing length 18-23 mm. Antennae weak bipectinate, head and thorax dark brown mixed with mossy green. Fore-wing broadly triangular, with pointed apex and corrugated outer margin, dorsum cilia reddish. Ground colour dark brown; wing pattern distinct, basal line twisted, forming a “3” shape. Reniform stigma large, green, terminally edged with yellow. The postmedial line is a fine, partly white-filled thin line, that is curved inwards around posterior edge of the cell. The inner half of marginal area broad and green bordered by the subterminal line at outer side. Hind-wing ground colour dark brown; discal spot faint; terminal area and cilia slightly darker than inner. Abdomen dark brown, distal segment covers in tufts.

**Male genitalia** (Fig. 2a). Valva elongated and slightly curved with a pointed setose apex and broad basal part. Harpe is strongly sclerotised, curved at the base, apical part bifurcated, forming a “C” shape. Juxta calabash-shaped. the aedeagus with a large, sclerotised, multispinose dorsal carinal plate and bicuspical ventral carinal extension. Vesica membranous tubular, with a wedge-shaped cornutus.

**Female.** The description is limited to the single incomplete female specimen that was collected. Wingspan 45 mm, fore-wing length 21.5 mm. Antennae filiform, wing pattern close to male.

**Figure 3.**
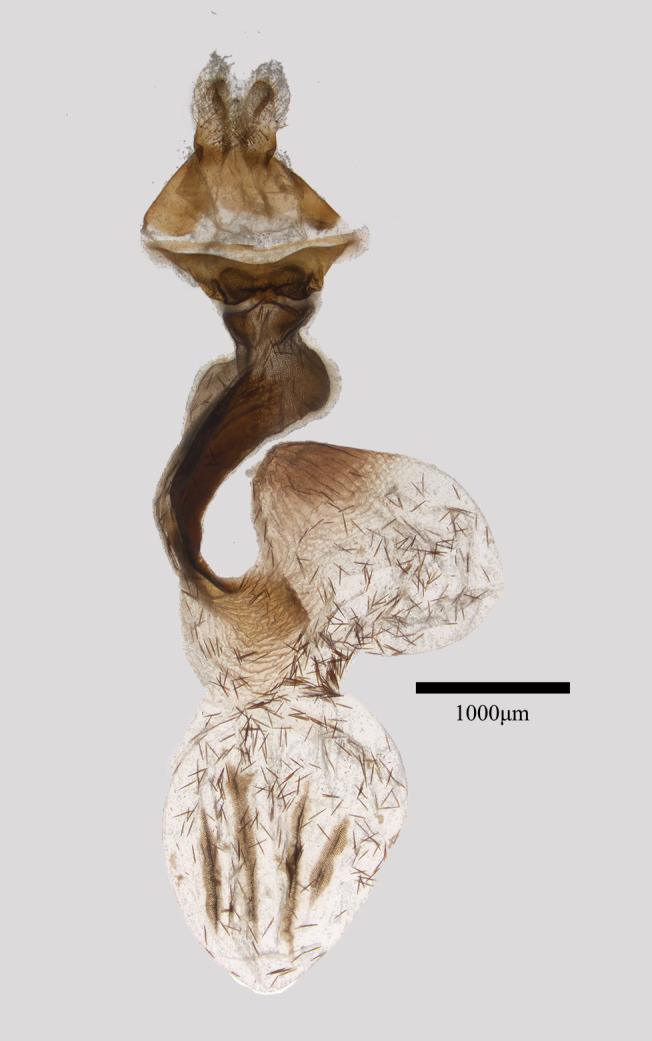
*Antivaleriaronkayorum* sp. nov., female, paratype.

**Female genitalia** (Fig. 3). Anal papillae short and weak. Ostium bursae small, trapezoidal, ventral plate smoothly sclerotised, the dorsal plate is much smaller, consisting of two scobinate hemispheres. Ductus bursae is long, granulosely sclerotised, with a strong sclerotised lobe. Cervix bursae is large egg-shaped, wrinkled and apically sclerotised. Corpus bursae is oval, with two long, one slightly short and one much shorter signum-stripes.

#### Diagnosis

The new species resembles *A.peregovitsi* Ronkay et al., 2010, but differs in the postmedial line curved inwards around posterior edge of the cell; the uncus is broader, the cucullus is more foot-shaped with a more pointed apex, the dorsal section of juxta is more slender, differently shaped distal part of harpe and a larger dentated plate of the dorsal carina; the dorsal plate of ostium bursae is much smaller.

#### Etymology

The species name is dedicated to Drs. Gábor Ronkay and László Ronkay in honour of their marvellous work on Noctuidae.

#### Distribution

**Figure 4.**
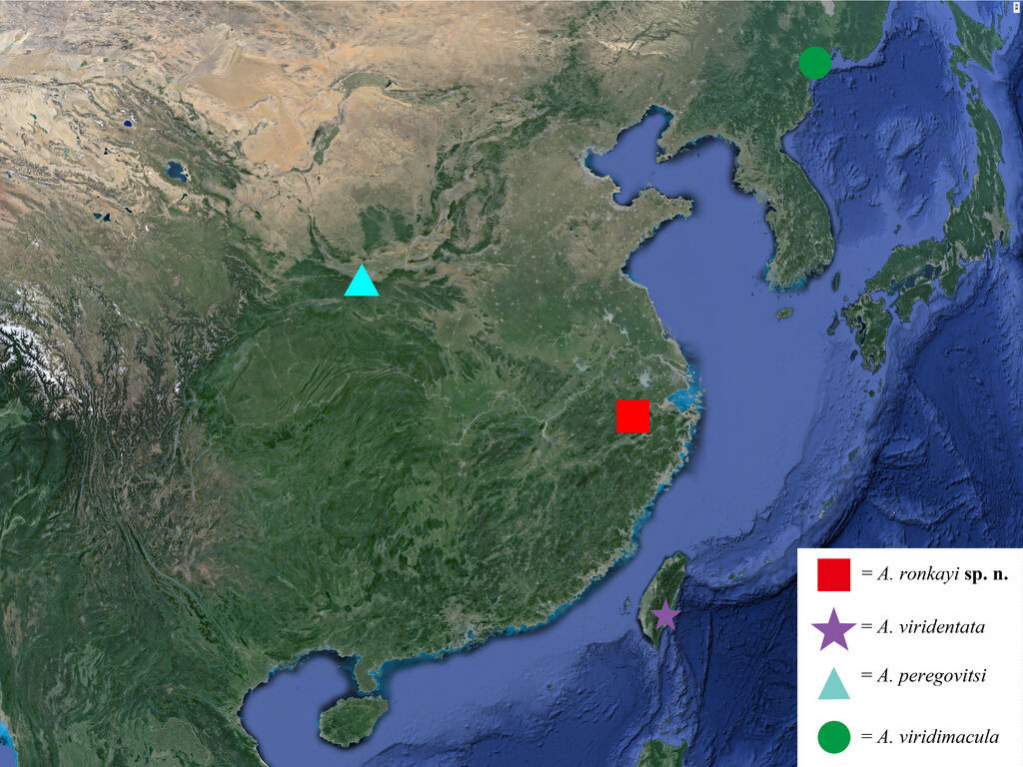
Type localities of *Antivaleria* spp.

China, Zhejiang (Chun’an Fig. 4).

## Identification Keys

### Key to species of *Antivaleria*

**Table:** 

1	Harpe apex non-bifurcated	*A.viridimacula*
–	Harpe apex bifurcated	2
2	The distal part of valva slender	*A.viridentata*
–	The distal part of valva broad	3
3	Valva rounded apically, harpe tip snake-head-like	*A.peregovitsi*
–	Valva pointed apically, harpe tip “C” shape	*A.ronkayorum* sp. nov.

## Supplementary Material

XML Treatment for Antivaleria ronkayorum

## Figures and Tables

**Figure 1a.**
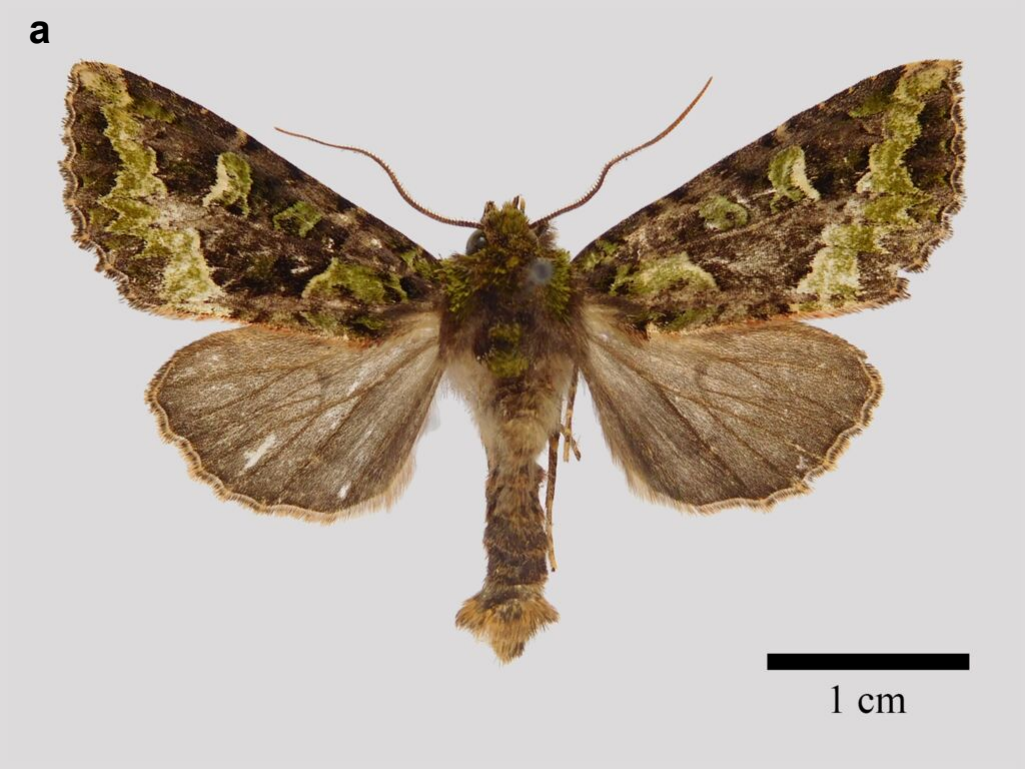
*Antivaleriaronkayorum* sp. nov., male, holotype;

**Figure 1b.**
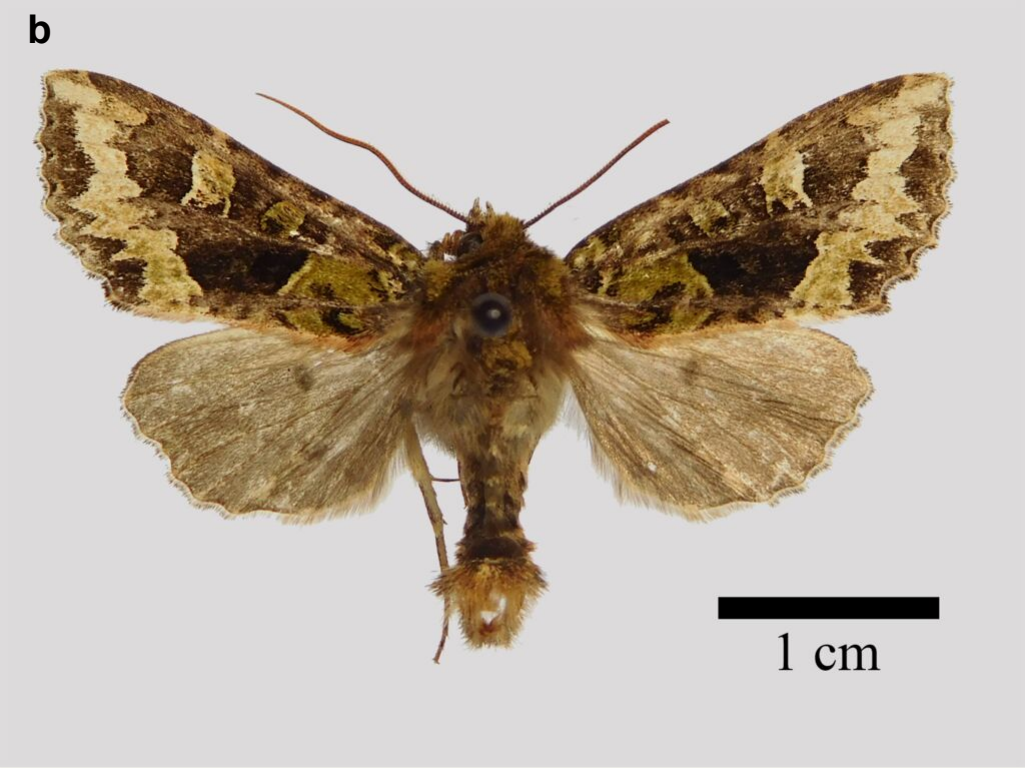
*Antivaleriaperegovitsi* Ronkay et al., 2010, male, Shaanxi.

**Figure 2a.**
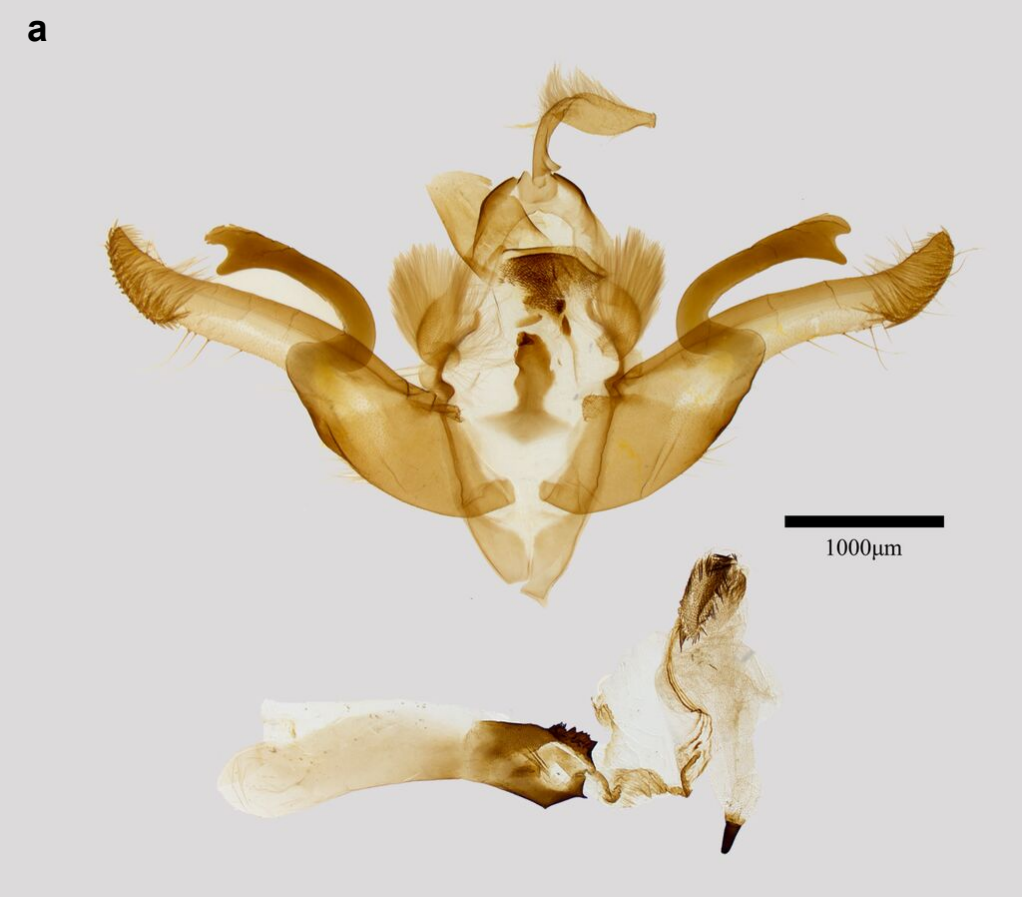
*Antivaleriaronkayorum* sp. nov., male, paratype;

**Figure 2b.**
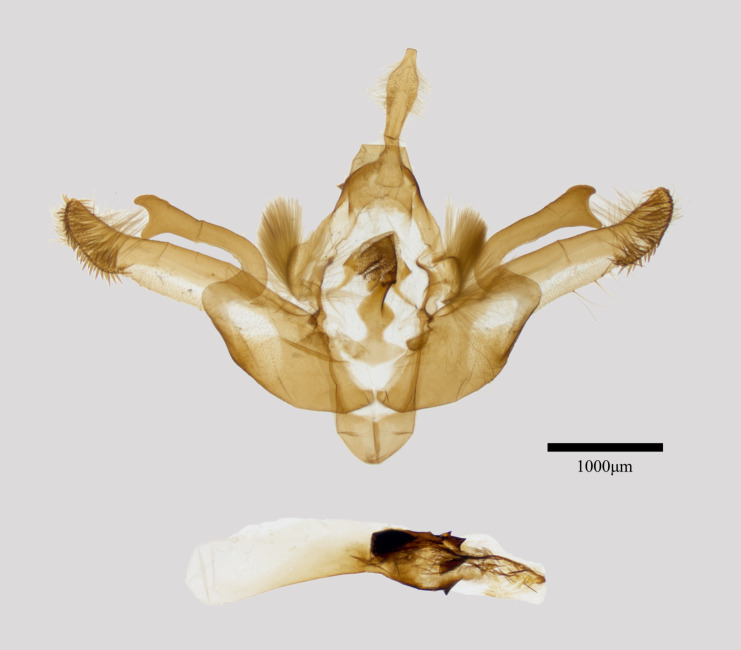
*Antivaleriaperegovitsi* Ronkay et al., 2010, male, Shaanxi.
